# Non-Coding RNA m6A Modification in Cancer: Mechanisms and Therapeutic Targets

**DOI:** 10.3389/fcell.2021.778582

**Published:** 2021-12-22

**Authors:** Da-Hong Chen, Ji-Gang Zhang, Chuan-Xing Wu, Qin Li

**Affiliations:** ^1^ Department of Clinical Pharmacy, Shanghai General Hospital, Shanghai Jiao Tong University School of Medicine, Shanghai, China; ^2^ Clinical Research Center, Shanghai General Hospital, Shanghai Jiao Tong University School of Medicine, Shanghai, China; ^3^ Department of General Surgery, Shanghai General Hospital, Shanghai Jiao Tong University School of Medicine, Shanghai, China

**Keywords:** m6A RNA modification, non-coding RNA, tumorigenesis mechanism, cancer therapy, epigenetics

## Abstract

Recently, N6-methyl-adenosine (m6A) ribonucleic acid (RNA) modification, a critical and common internal RNA modification in higher eukaryotes, has generated considerable research interests. Extensive studies have revealed that non-coding RNA m6A modifications (e.g. microRNAs, long non-coding RNAs, and circular RNAs) are associated with tumorigenesis, metastasis, and other tumour characteristics; in addition, they are crucial molecular regulators of cancer progression. In this review, we discuss the relationship between non-coding RNA m6A modification and cancer progression from the perspective of various cancers. In particular, we focus on important mechanisms in tumour progression such as proliferation, apoptosis, invasion and metastasis, tumour angiogenesis. In addition, we introduce clinical applications to illustrate more vividly that non-coding RNA m6A modification has broad research prospects. With this review, we aim to summarize the latest insights and ideas into non-coding RNA m6A modification in cancer progression and targeted therapy, facilitating further research.

## 1 Introduction

In eukaryotic cells, some non-coding ribonucleic acids (ncRNAs), such as microRNAs (miRNAs), long non-coding RNAs (lncRNAs), and circular RNAs (circRNAs), usually do not encode proteins but perform their respective biological functions at the RNA level. Generally, ncRNAs have long been thought to be post-transcriptional regulators of gene expression, but RNA modifications have rendered it possible for ncRNAs to generate new regulatory effects on gene expression. N6-methyl-adenosine (m6A), methylated at the N6 position of adenosine, is a reversible epigenetic RNA modification that modulates splicing, degradation, and other biological processes of RNAs (Fazi and Fatica 2019; Zhou et al., 2020)**.** During the process from DNA to RNA, adenylate undergoes methylation modification at the sixth position under the action of methyltransferase (“writer”) METTL3/14, WTAP, KIAA1429, RBM15, and ZC3H13. Next, the bases are demethylated by demethyltransferase (“eraser”) FTO and ALKBH. Finally, these methylated RNA base sites require specific enzymes (“readers”) to accomplish the purpose of embellishing ncRNAs (Sun et al., 2019). The molecular mechanisms of m6A modification involved in the regulation of ncRNA gene expression have been reported previously (Erson-Bensan and Begik 2017; Fazi and Fatica 2019; He et al., 2020; Zhang et al., 2020d), and more interestingly, the abundance of m6A modification and the expression of m6A regulators are also regulated by ncRNAs.

**FIGURE 1:**
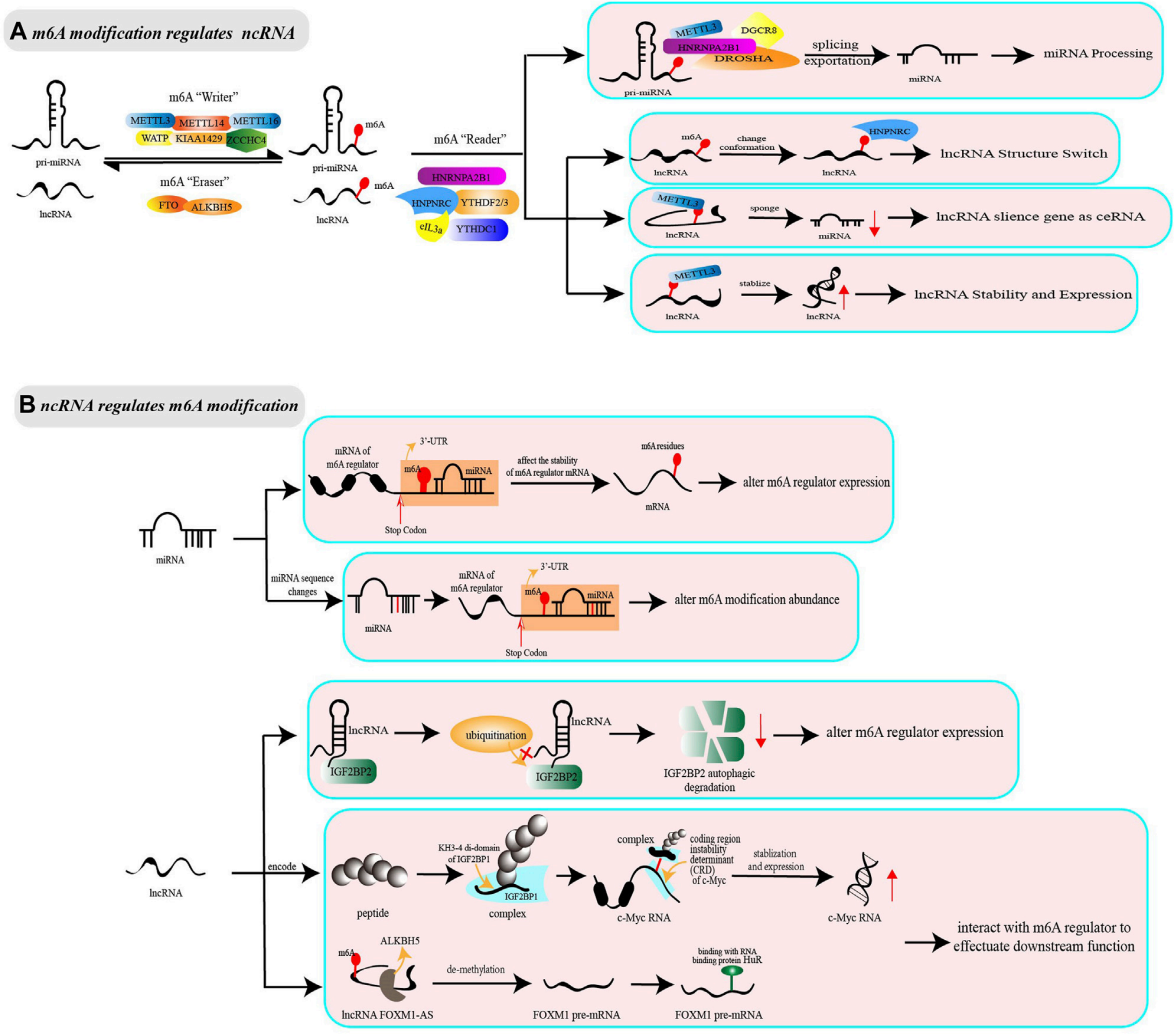
The interaction between non-coding RNA and m6A modification. The adenosine(A) bases reside in non-coding RNA could be methylated by methyltransferase complex (“Writer”) comprised of METTL3/METTL14/WATP and other regulator cofactors. The non-coding RNA with m6A modification resides could by recognized by m6A binding proteins (“Reader,” e.g. NRNPA2B1, HNRNPC, YTHDF) to effectuate downstream functions, or be reversibly erased by demethyltransferase (“Eraser,” e.g. FTO, ALKBH5). **(A)** m6A modification regulates ncRNA. During the processing from pri-miRNA to miRNA, the presence of m6A modification can regulate the splicing and exportation, facilitating the maturation of miRNA. Besides, m6A modification can play extraordinary complex and diversity roles in lncRNA. The lncRNA marked m6A writer proteins could change conformation to bind to proteins, leading to that m6A modification is regarded as lncRNA structure switch and facilitate the RNA-protein interaction. Secondly, the lncRNA which is methylated by m6A writer proteins have the effect on sponging miRNA as ceRNA, greatly augmenting the RNA-RNA interaction. For lncRNA itself, m6A modification could regulate transcript stabilization and expression level to activate or participate in subsequent functions. **(B)** ncRNA regulates m6A modification. Non-coding RNA could regulate m6A modification from the perspective of expression level and function. MiRNA integrate into the mRNA 3′-UTR region of m6A regulator cofactors, which alters the expression level of m6A regulators and indirectly influences on the abundance of m6A modification. Differently, lncRNA can alter the expression level of m6A regulators by affecting the stability and degradation of mRNA. Notably, lncRNA or RNA-binding regulatory peptide that is encoded by lncRNA (e.g. LncRNA LINC00266-1) has the capability to bind with m6A regulator to strengthen m6A recognition, therefore affecting the stabilization and expression of the downstream target mRNA. Anti-sense lncRNA could recruit m6A eraser proteins to decrease m6A abundance on the sense mRNA to active downstream effects (e.g. lncRNA FOXM1-AS and m6A eraser ALKBH5).

The genes encoding miRNA are transcribed as long transcripts, called primary miRNA (pri-miRNA) in the nucleus. Pri-miRNA undergoes nuclear cleavage mediated by Drosha-DGCR8 complex, to form precursor miRNA (pre-miRNA), which is then transported to cytoplasm via Exportin-5 and sheared by RNase Ⅲ endonuclease Dicer to short RNA fragments, namely miRNA (Vishnoi and Rani 2017). Studies have shown that pri-miRNAs methylated by m6A are more likely to be identified by molecules that are responsible for miRNA maturation, thus accelerating the initiation, splicing, transportation and other processes of miRNA biogenesis (Alarcón et al., 2015a; Alarcón et al., 2015b; Chen et al., 2020c) (see Figure 1A). For example, the m6A “writer” METTL3, marked in pri-miRNA, has been reported to interact with DGCR8 and to positively promote the splicing and maturation of miR-25 (Alarcón et al., 2015b), miR-143-3p (Wang et al., 2019a), pri-miR-221/miR-222 (Han et al., 2019a), pri-miR-1246 (Peng et al., 2019), and miR-25-3p (Zhang et al., 2019a). Further, the m6A “reader” HNRNPC can directly bind pri-miR-21 to increase miR-21 expression (Park et al., 2012), whereas HNRNPA2B1 interacts with lncRNA LINC01234 to indirectly modulate miR-106b-5p maturation (Chen et al., 2020d). Inconsistent with the above findings, a study has shown that HNRNPA2B1 overexpression downregulates miR-29a-3p, miR-29b-3p, miR-222, and inversely upregulates miR-1266-5p, miR-1268a, and miR-671-3p (Klinge et al., 2019). Notably, the methyltransferase NSun2 blocks the splicing of pri-miR-125b2 and interferes with miR-125b cleavage, which is in contrast to the action of methyltransferase METTL3/14 (Yang et al., 2015; Yuan et al., 2014). These data suggest the extraordinary complexity and diversity of m6A modification in miRNA biosynthesis.

LncRNA is a kind of ncRNA with a length of more than 200 nucleotides, but was initially considered as “transcriptional noise” because its biological functions were unknown (Yang et al., 2020b). With further research, increasing associated machineries of lncRNA have been found. The current studies indicate that m6A modification can act as a structural “switch” to change the conformation of lncRNA, participate in the ceRNA model for silencing miRNA or affect the stability and expression of lncRNA (see Figure 1A). Specifically, m6A-modified lncRNA is demonstrated to have hairpins that are more suitable for conformation in the RNA-HNRNPC complex, suggesting that m6A modification acts as a “switch” to trigger the combination of lncRNA and HNRNPC (Liu et al., 2015; Zhou et al., 2016). In addition, m6A modification can facilitate lncRNA to act as endogenous RNAs (ceRNAs) and then sponge downstream miRNA by improving the stability of lncRNA transcript or reducing RNA degradation (Jin et al., 2019; Wang et al., 2020a). Notably, m6A modification contributes to the function of lncRNAs, as well as affects their expression level. It is reported that YTHDF1 knockdown downregulates LINC00278-sORF1 translation without changing the m6A modification level. METTL3, METTL14, and WTAP knockdown reduces the m6A modification level and LINC00278-sORF1 translation, while ALKBH5 increases them (Wu et al., 2020).

The interaction between m6A modification and ncRNAs is reciprocal, which means that ncRNAs can also regulate m6A modification in biological processes. Some miRNAs can target the mRNA of m6A regulators and integrate into the 3′-UTR region of mRNA to alter their stability and expression, thus indirectly affecting m6A modification abundance. Further, lncRNA can affect the stability and degradation of m6A-related enzymes or combine with them to form complexes, thus facilitating a regulatory effect on the downstream target mRNA of m6A regulators (see Figure 1B). For example, miR-33a, miR-600, miRNA let-7g, miR-744-5p, and miRNA-145 are shown to decrease the expression of METTL3, HNRNPC or YTHDF2 at both the mRNA and protein levels (Cai et al., 2018; Du et al., 2017; Kleemann et al., 2018; Wei et al., 2019; Yang et al., 2017). Furthermore, the IGF2BP2 mRNA is regulated by lncRNA LINRIS, which is not only responsible for maintaining IGF2BP2 mRNA stability but also prevents its degradation (Wang et al., 2019b). Except for changing the expression level of m6A regulators, ncRNA can change m6A abundance via a sequence-dependent manner. For example, the decrease of endonuclease Dicer leads to aberrant miRNA expression, and modulates the binding of m6A-related enzymes to mRNAs that contain miRNA binding sites, thus downregulating the abundance of m6A modification without affecting the expression of m6A regulators, METTL3, FTO, and ALKBH5 (Chen et al., 2015).

So far, several studies have enriched our understanding of the interactions between ncRNA and m6A modification, and indicated that aberrant expression of m6A regulators and m6A modification on ncRNAs can alter normal biological processes. Notably, this abnormal biological change caused by m6A modification makes ncRNAs involved in tumorigenesis and cancer progression, associating with cell proliferation and apoptosis, invasion and metastasis, cell stemness, drug resistance and other mechanisms that enhance the malignancy of cells and the difficulty of cancer therapies. Therefore, the role of ncRNA m6A modification in various cancers is worthy of further study to better understand the related mechanisms, contributing to provide insights for early cancer diagnosis, outcome prediction and cancer treatment strategies. In this review, we discuss the relationship between ncRNA m6A modification and cancer progression from the perspective of various cancers. In particular, we focus on important mechanisms in tumour progression and introduce potential clinical applications to illustrate more vividly that ncRNA m6A modification has broad research prospects in cancer. We aim to summarize the latest insights into ncRNA m6A modification in cancer progression and targeted therapy, facilitating further research.

### 2 Functions of ncRNA m6A Modification in Different Types of Cancer

#### 2.1 Lung Cancer and ncRNA m6A Modification

Lung cancer, also known as primary bronchial lung cancer, refers to malignant tumour originating in bronchial mucous epithelium or alveolar epithelium, and is mainly classified into adenocarcinoma, squamous cell carcinoma, large cell carcinoma and small-cell lung cancer. Due to the huge difference between small cell carcinoma and other types in biological behaviour, treatment prognosis and other aspects, lung cancer other than small cell lung cancer is collectively referred to as non-small-cell lung cancer (NSCLC) (Yousef and Tsiani 2017; Inamura 2018). During the progression of NSCLC, LINC01234 is reported to be an oncogenic lncRNA that interacts with m6A “reader” HNRNPA2B1. Overexpression of LINC01234 combined with HNRNPA2B1 recruits DGCR8 and facilitates the processing of several miRNA precursors, including pri-miR-106b. Interestingly, activated c-Myc by miR-106b-5p can bind to the LINC01234 promoter to create a positive feedback loop (Chen et al., 2020d). Contrast to interaction, METTL3 accelerates the splicing of the precursor and generates mature miR-143-3p, regulating the expression of oncogene miR-143-3p (Wang et al., 2019a). Similarly, the miR-107/LAST2 axis is regulated by the m6A “eraser” ALKBH5 in an HuR-dependent manner to decrease YAP activity and inhibit tumour growth (Jin et al., 2020); lncRNA MALAT1 is stabilized by the METTL3/YTHDF1 complex and its RNA level is increased with high levels of m6A modification (Jin et al., 2019). METTL3 has been shown to promote the expression of some crucial oncoproteins and facilitate tumour proliferation, apoptosis, and invasion in human lung cancer (Du et al., 2017). However, miR-600 and miR-33a inhibit the expression of METTL3, reversing its positive effects on NSCLC progression and playing the role of tumour suppressor genes (Du et al., 2017; Wei et al., 2019). In summary, the effect of m6A modification on cancers may be completely opposite under the regulation of ncRNAs, suggesting the relationship between ncRNA and m6A modification is complex and variable in cancer progression.

### 2.2 Liver Cancer and ncRNA m6A Modification

Primary carcinoma of the liver has multifactorial pathogenesis and is a malignant tumour occurring in hepatocytes or intrahepatic bile duct epithelial cells, including hepatocellular carcinoma (HCC), hepatobiliary carcinoma and hepatic sarcoma (Gao et al., 2020). HCC occurs in liver cells and is the main histological subtype of the hepatic malignancy (accounting for more than 90% cases of primary carcinoma of the liver). Overwhelming evidence has proved the regulatory roles of ncRNAs related to liver carcinogenesis, and their relationship can be summed up as “frenemy” which is means a kind of love-hate relationship (Wong et al., 2018). The miR-186, which plays the role of anti-hepatoblastoma, is weakly expressed in HCC tissues, and its overexpression inhibits cell aggressive characteristics; however, the increase of its direct target METTL3 reverses the inhibitory effect (Cui et al., 2020). In addition, another study has shown that METTL3 can improve the expression of c-Myc by increasing m6A modification, whereas miR-338-5p inhibit the expression of METTL3 to interfere with the lung cancer progression (Wu et al., 2021). The m6A “writer” METTL14 positively modulate pri-miRNA-126 maturation by interacting with DGCR8 to suppress the metastatic potential of HCC cells. Similar to the interaction of miR-186 and METTL3, the overexpression of pri-miRNA-126 inversely inhibits the repressing effect of METTL14 (Ma et al., 2017).

Thus, hostility alters their normal biological functions, while the friendship represents a positive correlation between ncRNAs and m6A regulators. With the help of m6A modification mediated by METTL3/METTL14, lncRNA LINC00958 (Zuo et al., 2020)and circ-SORE (Xu et al., 2020a)are upregulated because of enhancement of transcript stability. The m6A “reader” IGF2BP1 plays an oncogenic role in HCC progression, but the decrease of lncRNA LIN28B-AS can deplete the expression of IGF2BP1-dependent mRNAs, such as IGF2 and Myc, thereby inhibiting HCC cell proliferation, migration, and invasion (Zhang et al., 2020c). In addition to influencing the expression of molecules, m6A modification and ncRNA also affect their binding to downstream molecules. KIAA1429 is a member of m6A methyltransferases, and GATA3 mRNA is a direct downstream target of KIAA1429-methylation, facilitating the degradation of GATA3. Strikingly, KIAA1429 preferentially induces GATA3 pre-mRNA in a targeted manner under the guidance of the antisense gene lncRNA GATA3-AS to maintain their roles in cancer progression (Lan et al., 2019). In addition, m6A modification targets the 3ʹ-UTR of YAP, which induces the repression effect of miR-582-3p, although whether the m6A regulator is primarily responsible for this essential part of the interaction is unclear until now (Zhang et al., 2018). The complexity of m6A modification and ncRNAs suggest that it is necessary to further study the mechanisms and effects of their crosstalk in liver cancer, thus using the stable and significant indicators to assist early diagnosis and treatment.

### 2.3 Gastric Cancer and ncRNA m6A Modification

Gastric cancer (GC) is a malignant tumour of digestive system occurring from gastric mucosal epithelium and glandular epithelium (Karimi et al., 2014), accounting for the second largest malignant tumour in China. In GC tissues, it is found that MEETL3 promotes the maturation of pri-miR-17-92 (Sun et al., 2020b), and m6A-modified motif-assisted miR-660 to reduce oncogene E2F3 activity (He and Shu 2019). Besides, METTL3 can upregulate the expression of target mRNA SEC62 by facilitating the stabilizing effect of IGF2BP1 on SEC62, but this positive regulation could be inhibited by miR-4429 (He et al., 2019). In contrast, lncRNA ARHGAP5-AS1 is responsible for recruiting METTLE3 to stabilise ARHGAP5 mRNA in the cytoplasm (Zhu et al., 2019). Unlike enhancing downstream target mRNA stability, METTL3 has been reported to combine with the m6A “reader” YTHDF2 to promote the degradation of PTEN mRNA and increase tumour malignancy, and subtly, the oncogenic lncRNA LINC00470 serves as an accelerator in this process (Yan et al., 2020). Many studies have reported that aberrant expressions of lncRNA (Sun et al., 2016), miRNA (Shin and Chu 2014), and circRNA (Shan et al., 2019) play important roles in GC progression and could be regarded as diagnostic/prognostic markers and chemotherapeutic tools. Compared with the complex change of m6A modification in liver cancer, most ncRNAs and protein genes related to m6A are upregulated in GC tissues, suggesting that the m6A-related risk score might be informative for risk assessment and prognostic stratification (Guan et al., 2020).

### 2.4 Bladder Cancer and ncRNA m6A Modification

Bladder cancer is a morbid malignancy of the urinary tract, and 95% of bladder tumors originate from epithelial tissue. Notably, the symptoms of bladder cancer mimic those of a urinary tract infection, increasing the diagnosis difficulty and delaying timely therapy (Li et al., 2019c). Accumulating evidence implies that the abnormal m6A modification of ncRNA is related to tumorigenicity and poor prognosis in bladder cancer. Oncogenic METTL3 facilitates DGCR8 to recognise pri-miRNA and accelerate the maturation of pri-miR221/222, which is targeted to PTEN (Han et al., 2019a). Differently from the direct regulation, the m6A “eraser” FTO regulates the expression of MALAT/miR-384/MAL2 axis by catalysing MALAT1 demethylation in an m6A-dependent manner, indicating the potential of FTO as a diagnostic and prognostic biomarker (Tao et al., 2021). Low expression of METTL14 and decreased global m6A abundance in bladder cancer tissue are also associated with the clinical severity and outcomes of bladder cancer. METTL14 knockout promotes the proliferation, self-renewal, metastasis, and initiation of tumour cells, while overexpression plays an adverse role (Gu et al., 2019). These m6A-related molecules may play important roles in the clinical diagnosis and treatment of bladder cancer in the future.

### 2.5 Breast Cancer and ncRNA m6A Modification

Breast cancer is an epithelial malignant tumour originating from the terminal ductal lobule unit of the breast. The breast epithelial cells have unlimited replicative potential and other malignant characteristics because of various carcinogenic factors (Nagini 2017). The hostile hypoxic microenvironment is a major reason for the rapid expansion of cancer cells. It has been reported that the lncRNA KB-1980E6.3 that is associated with hypoxia can recruit m6A “reader” IGF2BP1 to stabilize c-Myc mRNA and enhance the malignant characteristics (Zhu et al., 2021). In endocrine-resistant LCC9 breast cancer cells, HNRNPA2B1 expression is higher than that in parental and tamoxifen-sensitive cells, and this increase alters the transcriptome and expression of miRNAs, because HNRNPA2B1 is a “reader” of the m6A mark in pri-miRNAs and is responsible for promoting DROSHA processing of pre-miRNAs (Klinge et al., 2019). Similarly, m6A methyltransferase METTL14 has been demonstrated to be significantly increased in breast cancer tissues with the function of reshaping miRNA profile of cancer cell lines. It has been verified that hsa-miR-146a-5p is a differentially expressed miRNA, modulated by METTL14-induced m6A modification, and thus affects the migration and invasion of breast cancer cells (Yi et al., 2020). In addition, METTL14 is regulated by the oncogenic lncRNA LNC942 to facilitate cell growth and cancer progression. Mechanistically, LNC942 recruits the METTL14 protein and elevated post-transcriptional METTL14-mediated m6A modification levels, thereby stabilising the expression of downstream targets (Sun et al., 2020a). In human epidermal growth factor receptor 2 (HER2)-positive breast cancer, it is reported that m6A “eraser” FTO inhibits miR-181b-3p and upregulates the expression of oncogenic ARL5B (Xu et al., 2020b). Overall, more extensive and in-depth researches on the aberrant m6A modification of ncRNA for the carcinogenesis and development of breast cancer are required.

### 2.6 Colorectal Cancer and ncRNA m6A Modification

Carcinoma of large intestine is a malignant gastrointestinal tract tumour that originates from large intestine mucous membrane epithelium and gland with a high metastasis and reoccurrence rate, including colon cancer and carcinoma of the rectum (Muller et al., 2016). Most researchers focus on the relationship between ncRNA m6A modification and metastasis of colorectal cancer (CRC). A novel lncRNA RP11-138 J23.1 (RP11) is demonstrated to positively regulate epithelial-mesenchymal transition (EMT) in CRC cells. Mechanistically, the expression of RP11 is upregulated in an m6A modification-dependent manner; then, the complex composed by RP11 and HNRNPA2B1 prevents Zeb1 degradation, triggering the dissemination of cancer cells (Wu et al., 2019). LncRNA GAS5 promotes YAP ubiquitin-mediated degradation, but YTHDF3 alleviates this effect via lncRNA GAS5 decay, confirming that YTHDF3 is not only a novel target of YAP but also a significant player in YAP signalling by facilitating lncRNA GAS5 degradation (Ni et al., 2019). Overexpression of METTL14 is also reported to correlate with the YAP pathway and can suppress CRC cell growth and metastasis via the miR-375/YAP1 pathway (Chen et al., 2020b). Besides, the expression of oncogene lncRNA XIST negatively correlates with YTHDF2 and METTL14 in CRC tissues because of the m6A modification (Yang et al., 2020c). In contrast, YTHDC1 recognises circNSUN2 and export it to the cytoplasm, eventually participating in cancer progression by forming the cir-cNSun2/IGF2BP2/HMGA2 complex (Chen et al., 2019a). In addition, the oncogene lncRNA LINC00460 combines with IGF2BP2 and upregulates the expression of HMGA1 mRNA and increase its stability, participating in cancer development (Hou et al., 2021). The m6A “reader” YTH protein family and IGF2BP2 have been widely reported in colorectal cancer, and therefore it is reasonable to speculate that the m6A “reader” may play an important role in the progression of colorectal cancer, which deserves deeply study.

### 2.7 Other Cancers and ncRNA m6A Modification

**FIGURE 2:**
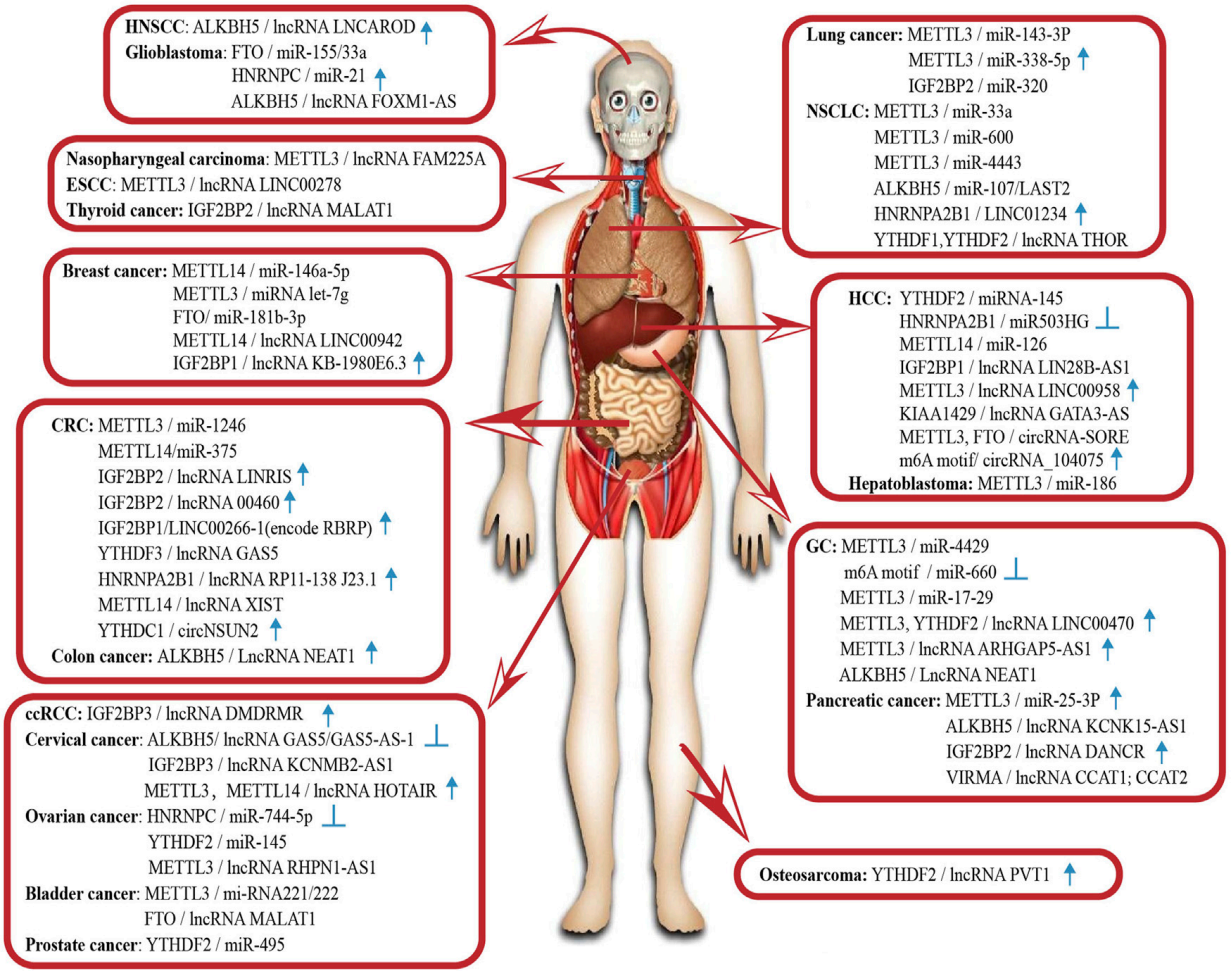
Abnormal m6A modification of non-coding RNA in human cancers. HNSCC, head and neck squamous cell carcinoma; ESCC, esophageal squamous cell carcinoma; HCC, hepatocellular carcinoma; CRC, colorectal cancer; NSCLC, non-small-cell lung carcinoma; GC, gastric cancer; ccRCC, clear cell renal cell carcinoma. The icons represent the ncRNAs that can be regarded as potential diagnostic and prognostic biomarkers. ↑ High expression is positively associated with poor prognosis. High expression is negatively associated with poor prognosis.

Abnormal m6A modification of ncRNAs is also associated with the progression of other cancers. In clear cell renal cell carcinoma, Gu et al. find a novel DNA methylation-deregulated and RNA m6A reader-cooperating lncRNA (DMDRMR), which interacts with IGF2BP3 to stabilize target genes and plays a carcinogenic role (Gu et al., 2021). In thyroid cancer, lncRNA MALAT1 competitively binds to miR-204, upregulates IGF2BP2 and enhances MYC expression in m6A-dependent manner, conferring a stimulatory effect on cancer progression (Ye et al., 2021). In osteosarcoma, ALKBH5 inhibits the degradation of lncRNA PVT1 and leads to the overexpression of lncRNA PVT1. Overexpressed PVT1 can suppress the binding of YTHDF2 with PVT1 to promote cancer cell proliferation (Chen et al., 2020a). This combination of the m6A “reader” and lncRNA, which affects cancer cell proliferation, also occurs in pancreatic cancer. IGF2BP2 and lncRNA DANCR have been reported to interact and promote the cancer stemness-like properties of pancreatic cancer. The adenosine at 664 of DANCR is modified by N6-methyladenosine, and IGF2BP2 serves as a reader for this structural change to stabilise DANCR RNA and promote cell proliferation (Hu et al., 2020). In pancreatic duct epithelial cells, oncogenic miR-25-3p is correlated with poor prognosis in patients with pancreatic cancer. One study has found that NF-κB-associated protein induced by m6A modification facilitates excessive pri-miR-25 maturation under the environment of cigarette smoke condensate; then it activates the oncogenic AKT-p70S6K signalling and provokes malignancy of pancreatic cancer (Zhang et al., 2019a). In head and neck squamous cell carcinoma, oncogenic LNCAROD affects the degree of tumour malignancy. METTL3-and METTL14-induced dysregulated m6A modification could account for the aberrant expression of LNCAROD and lead to the malignant behaviour of tumour cells (Ban et al., 2020). Moreover, Zheng et al. propose that m6A is highly enriched in oncogenic lncRNA FAM225A and enhances RNA stability, contributing to cell proliferation, migration, invasion, and metastasis (Zheng et al., 2019). Similarly, m6A modification improves the transcript stability of lncRNA RHPN1-AS1 and upregulates its expression in epithelial ovarian cancer tissues, facilitating RHPN1-AS1 to act as an oncogene (Wang et al., 2020a). In glioblastoma, it has been found that 13 central m6A methylation regulators are associated with the clinical and molecular phenotype, suggesting that m6A regulators are important participants in malignant progression (Du et al., 2020b). A more specific example is HNRNPC, which directly binds to pri-miR-21 and promotes its expression, playing a role in the metastatic potential of the glioblastoma cell lines. The experimental results show that the expression levels of HNRNPC are higher in highly aggressive cancer cells and are elevated along with the brain tumour grade (Park et al., 2012). In the cancers listed above, the abundance of m6A modification and the enzymes involved in it can affect ncRNA maturation and activation and change their biological functions, which in turn can impact the progression of various cancers (see Figure 2).

## 3 Mechanisms of ncRNA m6A Modification in Cancer Progression

**FIGURE 3:**
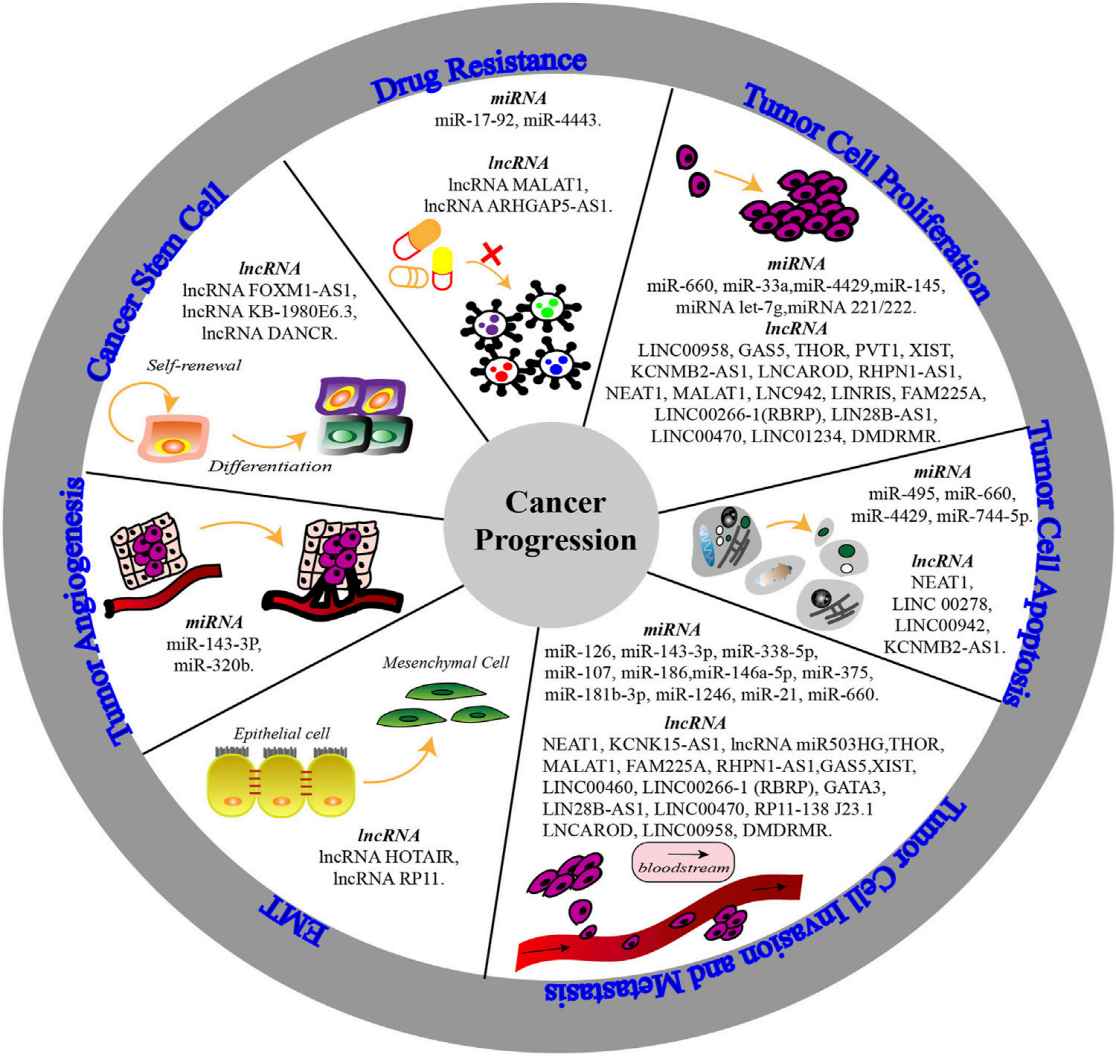
The mechanisms of non-coding RNA (miRNA & lncRNA) m6A modification involved in cancer progression. The biological functions of non-coding RNAs are altered by abnormal m6A modification and the interaction with m6A regulators. Thereby, non-coding RNAs participate in tumor cell proliferation, apoptosis, invasion and metastasis, epithelial-mesenchymal transition (EMT), tumor angiogenesis, cancer stemness and drug resistance to affect cell characteristics and cancer progression.

The ncRNA and m6A regulators show significant changes in different cancers owing to their interactions, which cause abnormal biological functions and thereby affect cancer progression (see Figure 3). Among urological cancers (Wang et al., 2020c), METTL3 is overexpressed in bladder cancer (Xie et al., 2020)and prostate cancer (Yuan et al., 2020), but shows low expression in kidney cancer (Tao et al., 2020). METTL14 is downregulated in kidney and bladder cancers, playing a tumour suppressive role. Surprisingly, even though METTL3 and METTL14 show different expression levels and functions in urological cancers, they both participate in cancer progression through cell growth- and cell death-related pathways (Tao et al., 2020). Therefore, understanding ncRNA m6A modification from the perspective of cancer progression mechanisms will provide more promising ideas and methods to interfere with cancer progression (see Table 1).

**TABLE 1:** The biological function and mechanism of ncRNAs and m6A modification involved in cancer progression.

Non-coding RNA	Cancer	Biological function and mechanism	References
miR-143-3P	lung cancer	Methylation facilitates miR-143-3p biogenesis and promotes brain metastasis *via* miR-143-3p/VASH1 axis	Wang et al. (2019a)
miR-338-5p	lung cancer	MiR-338-5p inhibits cell growth and migration *via* inhibition METTL3/c-Myc pathway	Wu et al. (2021)
miR-320b	lung cancer	miR-320b suppresses HNF4G and IGF2BP2 expression to inhibit angiogenesis and tumor growth	Ma et al. (2021)
miR-33a	NSCLC	MiR-33a attenuates cell proliferation by reducing the expression of METTL3	Du et al. (2017)
miR-107	NSCLC	ALKBH5 regulates miR-107/LATS2 axis to inhibit YAP, thus inhibiting tumor growth and metastasis	Jin et al. (2020)
miR-600	NSCLC	MiR-600 downregulates METTL3 expression to induce migration, proliferation and apoptosis	Wei et al. (2019)
miR-4443	NSCLC	Exosomal miR-4443 facilitates tumor growth and promotes cisplatin resistance *via* METTL3	Song et al. (2021)
lncRNA THOR	NSCLC	YTHDF1/2 regulate the stability of lncRNA THOR, strengthening cell proliferation and metastasis	Liu et al. (2020a)
lncRNA MALAT1	NSCLC	MALAT1 is stabilized by METTL3/YTHDF3 complex and sponges miR-1914-3p to promote invasion, metastasis and resistance	Jin et al. (2019)
lncRNA LINC01234	NSCLC	LINC01234 interacts with HNRNPA2B1 to enhance cell growth *via* miR-106b/CRY2/c-Myc axis	Chen et al. (2020d)
miR-186	hepatoblastoma	METTL3/miR-186 axis contributes to migration and invasion *via* the Wnt/β-catenin signaling pathway	Cui et al. (2020)
miR503HG	HCC	MiR503HG promotes HNRNPA2B1 degradation and inhibits migration *via* NF-κB signaling pathway	Wang et al. (2018)
miR-126	HCC	Methylation facilitates miR-126 maturation and alters cell metastatic capacity	Ma et al. (2017)
lncRNA LIN28B-AS1	HCC	LIN28B-AS1 downregulates IGF2BP1-dependent mRNAs, inhibiting proliferation, migration and invasion	Zhang et al. (2020c)
lncRNA LINC00958	HCC	Methylated LINC00958 sponges miR-3619-5p to upregulate HDGF, facilitating cell proliferation, motility and lipogenesis	Zuo et al. (2020)
lncRNA GATA3	HCC	GATA3-AS participates in the binding of KIAA1429 and GATA3, relating with tumor growth and metastasis	Lan et al. (2019)
circRNA-SORE	HCC	CircRNA-SORE induces sorafenib resistance *via* miR-103a-2-5p/miR-660-3p and Wnt/β-catenin pathway	Xu et al. (2020a)
circRNA_104075	HCC	CircRNA_104075 stimulates YAP-dependent tumorigenesis and cell proliferation through m6A modification	Zhang et al. (2018)
miR-4429	GC	MiR-4429 inhibits METTL3 to repress SEC62, preventing proliferation and facilitating apoptosis	He et al. (2019)
miR-660	GC	Ectopic expressed miR-660 directly binds to E2F3 and realizes anti-proliferation effect via the m6A motif	He and Shu (2019)
miR-17-92	GC	METTL3 facilitates the maturation of miR-17-92, decreases the resistance to mTOR inhibitor everolimus	Sun et al. (2020b)
lncRNA ARHGAP5-AS1	GC	lncRNA ARHGAP5-AS1 facilitates ARHGAP5 methylation, accelerating the chemotherapeutic resistance	Zhu et al. (2019)
lncRNA LINC00470	GC	LINC00470 decrease PTEN expression via m6A regulators, promoting proliferation, migration and invasion	Yan et al. (2020)
lncRNA NEAT1	GC	ALKBH5 and NEAT1 influences the expression of EZH2 and thus affects invasion and metastasis	Zhang et al. (2019b)
miR-146a-5p	breast cancer	METTL14 modulates hsa-miR-146a-5p expression, affecting migration and invasion of cancer cells	Yi et al. (2020)
miR-181b-3p	breast cancer	The FTO/miR-181b-3p/ARL5B signaling pathway regulates cell migration and invasion	Xu et al. (2020b)
miRNA let-7g	breast cancer	The loop of HBXIP/let-7g/METTL3/HBXIP related with cell proliferation	Cai et al. (2018)
lncRNA LINC00942	breast cancer	LNC942 increases methylation level, elevates cell proliferation and inhibits cell apoptosis	Sun et al. (2020a)
lncRNA KB-1980E6.3	breast cancer	LncRNA KB-1980E6.3 maintains CSC stemness via interacting with IGF2BP1 to facilitate c-Myc stability	Zhu et al. (2021)
miR-1246	CRC	Upregulated METTL3 facilitates migration and invasion via miR-1246/SPRED2/MAPK signaling pathway	Peng et al. (2019)
miR-375	CRC	METTL14 suppresses cell growth via the miR-375/YAP1, inhibits cell migration and invasion through the miR-375/SP1 pathway	Chen et al. (2020b)
lncRNA LINRIS	CRC	LINRIS stabilizes IGF2BP2 and prevents its degradation, promoting the aerobic glycolysis and proliferation	Wang et al. (2019b)
lncRNA GAS5	CRC	LncRNA GAS5 promotes YAP degradation, but YTHDF3 alleviates this effect via lncRNA GAS5 decay	Ni et al. (2019)
lncRNA LINC00460	CRC	LncRNA LINC00460 combines with IGF2BP2 to increase HMGA1, facilitating invasion and metastasis	Hou et al. (2021)
lncRNA RP11-138 J23.1	CRC	The m6A modification upregulates RP11 to promote EMT, migration, invasion and enhance liver metastasis	Wu et al. (2019)
lncRNA XIST	CRC	Methylation decrease enhances the expression of XIST, increasing tumorigenicity and metastasis	Yang et al. (2020c)
LINC00266-1 (RBRP)	CRC	The LINC00266-1-encoded RBRP peptide promotes tumorigenesis and metastasis via IGF2BP1/c-Myc	Zhu et al. (2020a)
Circ-NSUN2	CRC	YTHDC1 recognizes cir-cNSun2 and facilitates invasion by forming the cir-cNSun2/IGF2BP2/HMGA2 complex	Chen et al. (2019a)
lncRNA NEAT1	colon cancer	ALKBH5 upregulates NEAT1 expression by demethylation, which leads to inhibit apoptosis and induce cell proliferation and migration	Guo et al. (2020)
miR-25-3p	pancreatic cancer	Methylation facilitates pri-miR-25 maturation to provoke malignant phenotypes via AKT-p70S6K pathway	Zhang et al. (2019a)
lncRNA KCNK15-AS1	pancreatic cancer	Demethylation KCNK15-AS1is related with cell motility	He et al. (2018)
lncRNA DANCR	pancreatic cancer	DANCR is stabilized by IGF2BP2. IGF2BP2 and DANCR promote cancer stemness-like properties	Hu et al. (2020)
lncRNA GAS5/GAS5-AS-1	cervical cancer	GAS5-AS1 regulates m6A modification of GAS5 to inhibit proliferation and metastasis	Peng et al. (2016)
lncRNA KCNMB2-AS1	cervical cancer	KCNMB2-AS1 sponges miR-130b-5p/miR-4294 to upregulate IGF2BP3, inhibiting apoptosis of cancer cells and inducing proliferation	Zhang et al. (2020d)
lncRNA HOTAIR	cervical cancer	LncRNA HOTAIR methylated by m6A upregulate EMT related-genes and increase aggressiveness	Peng et al. (2016)
lncRNA RHPN1-AS1	epithelial ovarian cancer	RHPN1-AS1 is stabilized by methylation, promotes cell proliferation and metastasis *via* miR-596/LETM1 and activates FAK/PI3K/Akt pathway	Wang et al. (2020a)
miR-744-5p	ovarian cancer	The overexpression of miR-744-5p decreases HNRNPC, relating with cell apoptosis	Kleemann et al. (2018)
miR-145	ovarian cancer	MiR-145 downregulates YTHDF2 expression and increase m6A levels, suppressing cell proliferation	Li et al. (2020a)
miRNA221/222	bladder cancer	METTL3 overexpressed cells, miRNA221/222 promotes tumor proliferation by regulating PTEN.	Han et al. (2019a)
lncRNA DMDRMR	clear cell renal cell carcinoma	LncRNA DMDRMR-mediated regulation of m6A-modified CDK4 by IGF2BP3 promotes cell proliferation and metastasis	Gu et al. (2021)
lncRNA FOXM1-AS	Glioblastoma	FOXM1-AS regulates FOXM1 expression to maintain tumorigenicity of glioblastoma stemness-like cells	Zhang et al. (2017)
miR-21	glioblastoma multiforme	HNRNPC controls the metastatic potential by regulating the expression of miR-21 and PDCD4	Park et al. (2012)
lncRNA PVT1	Osteosarcoma	ALKBH5 upregulates PVT1 to suppress its binding with YTHDF2, promoting tumor proliferation	Chen et al. (2020a)
miR-495	prostate cancer	KDM5A/miRNA-495/YTHDF2/m6A-MOB3B axis is associated with cancer cell apoptosis	Du et al. (2020a)
lncRNA CCAT1/CCAT2	prostate cancer	M6A “reader” VIRMA downregulation attenuates the aggressive phenotype by overall reduction of m6A-levels decreasing stability and abundance of oncogenic lncRNA CCAT1 and lncRNA CCAT2	Barros-Silva et al. (2020)
lncRNA FAM225A	nasopharyngeal carcinoma	FAM225A with highly enriched m6A modification promotes tumorigenesis and metastasis *via* miR-590-3p/miR-1275/ITGB3	Zheng et al. (2019)
lncRNA LNCAROD	HNSCC	Methylation stabilizes LNCAROD and promotes cancer progression via HSPA1A/YBX1, associating with cell proliferation and mobility	Ban et al. (2020)
lncRNA LINC00278	ESCC	LINC00278 modified by m6A encodes a micropeptide YY1BM, whose downregulation upregulates eEF2K expression, disrupts negative regulation of the AR signaling pathway and inhibits cell apoptosis	Wu et al. (2020)
lncRNA MALAT1	thyroid cancer	MALAT1 upregulates IGF2BP2 and enhances Myc expression by competitively binding to miR-204, conferring a stimulatory effect on proliferation, migration, invasion and cell apoptosis	Ye et al. (2021)

### 3.1 Tumour Cell Proliferation

Many studies have reported that m6A modification of ncRNAs influences tumorigenesis and cell proliferation; for example, ectopic expression of miR-660 directly binds to oncogene E2F3 to affect cell proliferation owing to the m6A motif in the region of E2F3 3ʹ-UTR (He and Shu 2019). The stabilisation of oncogene SEC62 mRNA could be enhanced by the m6A modification of METTL3 and IGF2BP1; however, it was demonstrated that miR-4429 targets and suppresses METTL3 to repress SEC62 and realise an anti-proliferative effect (He et al., 2019). In addition, the overexpression of miR-145 inhibits cancer cell proliferation, while this suppression is impaired by the overexpression of YTHDF2, resulting in a crucial crosstalk between miR-145 and YTHDF2 by forming a double-negative feedback loop (Li et al., 2020a). Carcinogenic lncRNA THOR has been reported to be related to m6A modification for the first time. YTHDF1 and YTHDF2 can regulate the stability of THOR by reading the m6A motifs, thereby strengthening cell proliferation and helping THOR to realise its oncogenic function (Liu et al., 2020a). In terms of the m6A “reader,” METTL3-induced m6A methylation reduces lncRNA RHPN1-AS1 degradation to help it serve as a ceRNA and activate the PI3K/AKT pathway (Wang et al., 2020a). Similarly, LNC942, lncRNA XIST and lncRNA FAM225A can affect cell proliferation and cancer progression under the m6A “writer”-mediated m6A modification (Sun et al., 2020a; Yang et al., 2020c; Zheng et al., 2019).

With the participation of m6A modification, both lncRNA THOR and lncRNA RHPN1-AS1, regarded as oncogenes, can promote tumorigenesis and cell proliferation, while it is worth noting that LINC00266-1 itself cannot promote tumorigenesis but RBRP encoded by it is a potential oncogene in colorectal cancer. LncRNA LINC00266-1 encodes a 71-amino acid peptide named “RNA-binding regulatory peptide” (RBRP), which binds to the RNA-binding proteins, including the m6A “reader” IGF2BP1. The existence and expression of natural endogenous RBRP has been verified in colorectal, breast, and ovarian cancers and nasopharyngeal carcinoma. RBRP, considered as a regulatory subunit of a m6A “reader,” strengthens m6A recognition by IGF2BP1 on targeted RNAs through the essential G19 residue and promotes tumorigenesis (Zhu et al., 2020a).

### 3.2 Tumour Cell Apoptosis

To investigate the function of miRNA m6A modification in cell apoptosis, regions upstream and downstream of miRNA 675 m6A modification sites in the H19 locus are mutated with a system of adenine base editors, showing that mutation facilitates cell apoptosis by downregulating the expression of H19 (Hao et al., 2020). Gu et al. (2018) also believed that m6A-modified miRNAs regulated the pathway closely related to cell apoptosis, as verified in arsenite-transformed cells. In prostate cancer, YTHDF2 is regarded as a target of miR-495 to recognise MOB3B m6A modification and inhibit its expression. At the same time, the miR-495 promoter interacts with overexpressed oncogenic KDM5A and restrains the expression of miR-495. It has been reported that the progression of prostate cancer can be motivated by the activation of the KDM5A/miRNA-495/YTHDF2/m6A-MOB3B axis by influencing the apoptosis of cancer cells (Du et al., 2020a). YTHDF2 is also considered as a direct target gene of miR-145 in epithelial ovarian cancer, and its overexpression enhances cell apoptosis (Li et al., 2020a). Besides, cigarette smoke decreases the m6A modification of LINC00278 and the translation of YY1BM encoded by LINC00278, and these dysregulations inhibit the apoptosis of cancer cells (Wu et al., 2020). In addition, overexpressed lncRNA KCNMB2-AS1 plays the role of ceRNA and upregulates the oncogene IGF2BP3 by sponging miR-130b-5p and miR-4294. IGF2BP3 binds to KCNMB2-AS1 m6A sites, forming a positive regulatory loop composed of KCNMB2-AS1 and IGF2BP3 to inhibit cell apoptosis and induce proliferation (Zhang et al., 2020e).

### 3.3 Tumour Cell Invasion and Metastasis

Some researchers have proposed a correlation between m6A and invasion, metastasis, such as the overexpression of FTO, which promotes the migration and invasion of GC cell lines (Xu et al., 2017). ALKBH5 is involved in mediating methylation reversal, and it is reported that ALKBH5-demethylated lncRNA NEAT1 is overexpressed in GC and colon cancer cells, promoting invasion and metastasis (Guo et al., 2020; Zhang et al., 2019b). Yang et al. also determined that the downregulation of ALKBH5 in colon cancer is associated with tumour inhibition. Overexpression of ALKBH5 restrains the invasion and metastasis of colon cancer cells (Yang et al., 2020a). In addition, ALKBH5 demethylates lncRNA KCNK15-AS1 and downregulates its expression in pancreatic cancer tissues, inhibiting cell motility and invasion (He et al., 2018). These interesting roles of ALKBH5 in different cancers warrant further study. The m6A modification level is significantly reduced in HCC tissue, especially in metastatic tissues. It was confirmed that the main factor is the downregulation of METTL14, which mediates miRNA maturation and alters the metastatic capacity of HCC (Ma et al., 2017). LncRNA miR503HG is also reduced in HCC tissues. Further investigation suggested that HNRNPA2B1 degradation is promoted by miR503HG, suppressing the NF-κB pathway and facilitating the invasion and metastasis of HCC cells (Wang et al., 2018). In other tumours, METTL3-methylated regulates the MALAT1-miR-1914-3p-YAP axis and increases YAP activity to enhance drug resistance and metastasis (Jin et al., 2019). METTL3-mediated methylation can also enhance the stability of lncRNA FAM225A, which regulates the expression of ITGB3 by binding to miR-590-3p and miR-1275 as ceRNA, and thus activates the FAK/PI3K/AKT signalling pathway and promotes the invasion and migration of cancer cells (Zheng et al., 2019). Moreover, the carcinogenic lncRNA RHPN1-AS1 and lncRNA THOR have also been found to be associated with cell viability and mobility by ectopic m6A modification, thereby affecting cancer cell invasion and metastasis (Liu et al., 2020a; Wang et al., 2020a).

### 3.4 Epithelial-Mesenchymal Transition

During epithelial-mesenchymal transition (EMT) progression, the loss of E-cadherin causes cells to acquire mobility and the capacity (Castosa et al., 2018; Ribatti et al., 2020). It has previously been reported that the decrease of METTL3 inhibited the lung cancer cell morphological conversion induced by TGF-β, and augmented the expression changes of EMT-related marker genes, indicating the importance of m6A modification in EMT (Wanna-Udom et al., 2020). As for ncRNA m6A modification, it has been proposed that m6A-methylated lncRNA HOTAIR could upregulate EMT genes and increase the aggressiveness of tumour cells (Peng et al., 2016). In addition, lncRNA RP11 was reported to positively regulate the migration, invasion, and EMT of colorectal cancer cells. It is believed that m6A methylation increases nuclear accumulation and participates in the upregulation of RP11, triggering the spread of colorectal cancer cells (Wu et al., 2019). By analysing the cancer cells and clinical samples, Kandimalla et al. established the RNAMethyPro-a gene expression signature, which is correlated with EMT-related prognosis genes (Kandimalla et al., 2019). The specific mechanisms between ncRNA m6A modification and EMT deserve further study.

### 3.5 Tumour Angiogenesis

Angiogenesis is regarded as a significant step in tumour development, and some of the genes associated with EMT and tumour angiogenesis are m6A target genes involved in tumorigenesis; they are very sensitive to m6A modification. Previously, RNA functional analysis confirm that METTL14 and ALKBH5 can regulate the expression of each other, inhibit the demethylation activity of YTHDF3, and induce aberrant m6A modification to play roles in tumour angiogenesis and metastasis (Panneerdoss et al., 2018). In lung cancer, the anti-cancer gene miR-320b downregulates the expression of IGF2BP2 and thymidine kinase 1 (TK1), thus suppressing angiogenesis and lung cancer growth (Ma et al., 2021). During the process of lung cancer brain metastasis, upregulated miR-143-3p promoted by METTL3-methylation facilitates miRNA splicing and biogenesis, interacts with VASH1 and increases invasion capability and angiogenesis (Wang et al., 2019a). In brain metastasis of breast cancer, transcripts associated with brain metastasis are enriched by YTHDF3 and promote cancer cells to interact with cells in the tumour microenvironment, benefiting angiogenesis and metastasis (Chang et al., 2020).

### 3.6 Cancer Stem Cells

Cancer stem cells maintain the vitality of the cellular population through self-renewal and infinite proliferation, which are essential for cancer initiation and metastasis. Experimental analysis has shown that ALKBH5 facilitates the proliferation and tumorigenicity of cancer stem cells by interacting with lncRNA FOXM1-AS (Zhang et al., 2017). Moreover, the identified m6A “reader” YTHDF2 can reduce the half-life of diverse m6A transcripts, including the tumour necrosis factor receptor (Tnfrsf2), leading to negative effects on the overall integrity of leukemic stem cell function. Under YTHDF2 depletion, Tnfrsf2 is upregulated to facilitate cell apoptosis in leukemic stem cells (Paris et al., 2019). In addition to leukemic stem cells, YTHDF2 is associated with the liver cancer stem cell phenotype (Zhang et al., 2020b). In breast cancer, a recent study showed that lncRNA KB-1980E6.3 can augment cancer cell self-renewal and maintain the stemness of cancer cells under a hypoxic microenvironment, indicating that targeting the lncRNA KB-1980E6.3/IGF2BP1/c-Myc axis may be a promising therapy for refractory hypoxic tumours (Zhu et al., 2021).

### 3.7 Drug Resistance

Drug resistance that develops during conventional drug therapy is one of the significant reasons for chemotherapy failure in cancer. As the main component of methylase involved in m6A modification, METTL3 has been widely reported to be associated with drug resistance. In NSCLC, the METTL3/YTHDF3 complex enhances the stability of lncRNA MALAT1, regulating the MALAT1-miR-1914-3p-YAP axis to increase YAP expression and induce drug resistance (Jin et al., 2019); METTL3, as a direct target gene of miR-4443 in tumour exosomes, is involved in regulating FSP1 expression and interferes with the ferroptosis induced by cisplatin, resulting in drug resistance (Song et al., 2021). Furthermore, in GC cells, METTL3 is recruited by lncRNA ARHGAP5-AS1 to stabilize ARHGAP5 mRNA and promotes chemoresistance (Zhu et al., 2019), and consistent with this study, Sun et al. find that elevated METTL3 and levels of m6A modification facilitate the maturation of pri-miR-17-92 to the miR-17-92 cluster, which activates the AKT/mTOR pathway. High levels of METTL3 and the miR-17-92 cluster then enhance the sensitivity of cancer cells to the mTOR inhibitor, everolimus (Sun et al., 2020b).

In tamoxifen-resistant LCC9 breast cancer cells, studies have determined that overexpressed m6A “reader” HNRNPA2B1 can upregulate and downregulate different miRNAs simultaneously and affect the downstream signalling pathways of these miRNAs. Transient overexpression of HNRNPA2B1 reduces the sensitivity of cancer cells to 4-hydroxytamoxifen and fulvestrant, suggesting the potential role of HNRNPA2B1 in endocrine-resistance (Klinge et al., 2019). Oncogene circRNA-SORE is upregulated in sorafenib-resistant HCC cells, acting as ceRNA to sponge miR-103a-2-5p and miR-660-3p, competitively activate the Wnt/β-catenin pathway, and induce sorafenib resistance (Xu et al., 2020a). However, further study shows that interfering the expression of circRNA-SORE has effects on improving sorafenib efficacy, which represents a promising pharmaceutical intervention targeted circRNA-SORE in sorafenib-treated HCC patients. Besides, recent studies have shown that depletion or inhibition of FTO suppresses the expression of immune checkpoint genes and attenuates the self-renewal ability of cancer stem cells, thus overcoming their immune escape (Su et al., 2020). Another study indicated that the m6A modification induced by ‘eraser’ FTO augments melanoma growth, whereas FTO inhibition increases the sensitivity of cancer cells to anti-PD-1 immunotherapy, suggesting that the immune checkpoint molecular inhibitors represented by PD-1/PD-L1 can play an important role with the help of FTO inhibitors (Yang et al., 2019). Although these two studies were based on the demethylation effect of FTO on mRNAs, it is reasonable to believe that FTO inhibitors can also inhibit ncRNA-related targets or signal pathways with the deepening of studies on the crosstalk of FTO and ncRNAs. These findings provide in-depth insights into chemoresistance and support the therapeutic potential of ncRNA m6A modification, especially in combination with existing drugs to target refractory malignant tumours.

## 4 Clinical Application of ncRNA m6A Modification in Cancers

### 4.1 m6A Regulators and Abnormally Modified ncRNAs as Potential Diagnostic and Prognostic Biomarkers

The interaction between ncRNAs and m6A modification suggests that they may provide new ideas for the clinical treatment of cancer and even become prognostic biomarkers or therapeutic targets for cancer therapy. Zhang et al. constructed an m6A-score model to quantify m6A modification patterns in cancers. By analysing tumour microenvironment phenotypes and 5-year survival rates in patients with low and high m6A-score subgroups, it was found that patients with low m6A-scores show significant therapeutic advantages, suggesting a crosstalk between m6A modification and tumour microenvironment diversity and complexity (Zhang et al., 2020a). In HCC tissues, m6A methyltransferases WATP and KIAA1429 are related to prognosis (Chen et al., 2019b; Liu et al., 2020b), and METTL3, YTHDF2, ZC3H13 and ALKBH5 are considered independent prognostic factors for overall survival; of these, only METTL3 is an independent prognostic factor for recurrence-free survival (Li et al., 2019b; Liu et al., 2021). In CRC, depletion of METTL14 is associated with poor prognosis (Chen et al., 2020b; Yang et al., 2020c), which is different from the methylated enzymes METTTL3 and KIAA1429 in liver cancer. Notably, m6A “reader” ALKBH5 is associated with TNM stage, tumour size, lymph node metastasis (Tang et al., 2020), and can be regarded as a prognostic indicator in colon cancer (Guo et al., 2020). ALKBH5 is also believed to be a novel biomarker and independent prognostic factor in pancreatic cancer and NSCLC (Tang et al., 2020; Zhu et al., 2020b). Further, studies have revealed that FTO expression is closely correlated with low differentiation, peritumoral lymphovascular invasion, lymph node metastasis and is positively correlated with TNM stage in HCC, GC, HER2-positive breast cancer and bladder cancer, which makes FTO a possible biomarker for diagnostic and prognostic purposes (Cui et al., 2020; Tao et al., 2021; Xu et al., 2017; Xu et al., 2020b). Notably, high expression of HNRNPC in glioblastoma enhances cell invasiveness by regulating the miR-21/PDCD4 axis, and the level of HNRNPC is reported to increase with the increasing grade of brain tumour (Park et al., 2012). Bioinformatics analysis and validation experiments further confirmed the value of high expression HNRNPC in the prognosis of GBM patients at the expression level (Wang et al., 2020b). Interestingly, Ying et al. have identified m6A “reader” HNRNPC genes in the Chinese population and discussed the relationship between single nucleotide polymorphisms and susceptibility to pancreatic cancer, providing a completely novel idea on studying the role of m6A modification in tumorigenesis (Ying et al., 2021). Besides, several studies have analyzed the m6A regulators expression differences and their correlations in normal tissue and tumour tissue based on the clinical data of TCGA and GTEx databases, and it is found that the differential expression of some m6A regulators are closely associated with the risk factors of prognosis in hematologic system tumours and endocrine-system-related tumours (Li et al., 2020b; Zhang et al., 2021). Based on TCGA, GTEx and GEO, a novel risk signature has been constructed to evaluate the relationship between m6A regulators and survival rate of patients in uterine corpus endometrial carcinoma and uterine carcinosarcoma, further inspiring us to combine the bioinformatics with the study of m6A modification (Zou et al., 2021). Taken together, focusing on the expression of m6A regulators and their prognostic value provides us with a potential experimental avenue. It will have good clinical significance to directly or indirectly measure the expression level of m6A, the degree of tumours malignancy and the prognosis of patients by using tumour samples of patients.

ncRNAs modified by abnormal m6A modification could also be regarded as biomarkers, but these ncRNAs show a high degree of specificity and difference in various cancers. A m6A-lncRNA co-expression network has been constructed in primary glioblastoma to identify four m6A-related prognostic lncRNAs: MIR9-3HG, LINC00900, MIR155HG, and LINC00515 (Wang et al., 2021). Using a similar approach, LINC00152, LINC00265, and nine other lncRNAs are documented as biomarkers for predicting the overall survival of lower-grade glioma patients (Tu et al., 2020). In HCC, high expressed circRNA_104075 acts as a ceRNA to sponge miR-582-3p and upregulate YAP expression. The significant changes in downstream pathways caused by circRNA_104075 render it possible to be a new diagnostic marker (Zhang et al., 2018). LncRNA00266-1-encoded peptides RBRP bind to m6A “reader” IGF2BP1 to facilitate tumorigenesis, and the patients with high level of RBRP display an unfavourable prognosis (Zhu et al., 2020a); high expression of LINC00958 also can independently predict poor overall survival in patients with HCC. The good news is to take advantage of the role of LINC00958 as a biomarker, Zuo et al. constructed a novel drug delivery system, a PLGA-based nanoplatform filled with si-LINC00958, with the advantages of controlled release, tumour targeting, and better safety and effectiveness (Zuo et al., 2020). Differently, as a prognostic indicator, lncRNA miR503HG is significantly decreased in HCC tissues, and is positively associated with overall survival and time to recurrence (Wang et al., 2018).

The same phenomenon occurs in GC and cervical cancer. High expression of lncRNA ARHGAP5-AS1 (Zhu et al., 2019)and lncRNA LINC00470 (Yan et al., 2020) is related to the poor prognosis of patients with GC, whereas low expression of miR-660 is strongly linked with a large tumour size, severe lymph node metastasis, advanced TNM stage, and poor outcome (He and Shu 2019). In cervical cancer, lncRNA HOTAIR and lncRNA GAS5 show opposite effects on forecasting the clinical states of patients, high expression of HOTAIR indicates a high degree of malignancy, whereas lncRNA GAS5 is downregulated in cancer tissues, indicating that low GAS5 expression suggests poor prognosis (Peng et al., 2016). The above examples show that under m6A modification, multiple ncRNAs with significant changes may appear in the same cancer, which makes them have great potential for future prognostic diagnosis. However, accuracy cannot be determined solely using existing studies, and is worth further experimental exploration.

Surprisingly, lncRNA LINRIS (Wang et al., 2019b), lncRNA RP11 (Wu et al., 2019), lncRNA LINC00460 (Hou et al., 2021), lncRNA NEAT1 (Guo et al., 2020), and circRNA NSUN2 (Chen et al., 2019a) are all upregulated in patients with CRC and poor overall survival, indicating their promising role as critical prognostic markers and therapeutic targets. In prostate cancer, lncRNA CCAT1 and lncRNA CCAT2 can be regarded as a group variable to independently predict the prognosis of patients (Barros-Silva et al., 2020). Further, evidence confirms that potential prognostic markers also exist in other cancers, such as LINC01234 in NSCLC (Chen et al., 2020d), miR-744-5p in ovarian cancer (Kleemann et al., 2018), lncRNA LNCAROD in head and neck squamous cell carcinoma (Ban et al., 2020), lncRNA PVT1 in osteosarcoma (Chen et al., 2020a), miR-25-3p (Zhang et al., 2019a)and lncRNA DANCR (Hu et al., 2020)in pancreatic cancer (see Figure 2). Notably, several recent studies have used data models to analyse differentially expressed ncRNAs in some cancers to identify potential prognostic or diagnostic markers, such as in ovarian cancer (Li et al., 2021), adrenocortical carcinoma (Jin et al., 2021), kidney renal clear cell carcinoma (Yu et al., 2021), and colorectal cancer (Zuo et al., 2021). This new approach can explore the relationship between ncRNAs and different cancers on a large scale, as well as provide ideas and directions for further molecular mechanism research. To effectuate the role of ncRNAs and m6A regulators as biomarkers, the remarkable relationship between the expression level of m6A and prognosis needs to be explored more specifically. Demonstrating or testing the changes in m6A levels based on existing data in various cancers to evaluate clinical significance maybe more convincing.

### 4.2 m6A Regulators as Drug Targets to Participate in Cancer Therapy

Increasing attention has been focused on m6A regulators in hope of finding new treatments. For instance, a study shows that the new sodium channel blocker MV1035 can disturb migration and invasion characteristics by targeting ALKBH5 in glioblastoma (Malacrida et al., 2020). The potential of ALKBH as a drug target has been demonstrated by screening ALKBH blockers from newly synthesised anthraquinone derivatives (Huang et al., 2015). As an important demethylase, FTO has been identified to play an oncogenic role in NSCLC, ovarian cancer, and acute myeloid leukemia, associating with cancer cell proliferation, cancer stem cell maintenance and other malignant characteristics (Huang et al., 2020a; Huang et al., 2019; Li et al., 2019a). In recent years, researchers have used FTO as a target to develop FTO inhibitors, hoping to make new breakthroughs in cancer treatment. It is well known that meclofenamic acid, R-2-hydroxyglutarate, and MO-I-500 inhibit the activity of FTO and display anti-cancer activity (Huang et al., 2015; Singh et al., 2016; Su et al., 2018). Huang et al. find that artificially developed FTO inhibitors FB23 and FB23-2 can directly bind to FTO, selectively inhibit the m6A demethylase activity of FTO, and significantly inhibit cell proliferation in human acute myeloid leukaemia (Huang et al., 2019). Su et al. screen out two small-molecule FTO inhibitors, CS1 and CS2, and showed strong anti-cancer effects in multiple types of cancers. According to a previous study, the self-renewal of leukaemia stem cells can be attenuated and the immune response can be reprogrammed by CS1/2 (Su et al., 2020). A recent trial of FTO inhibitors shows that the ethylester form of meclofenamic acid (MA2) can negatively regulate Myc-miR-155/23a cluster-MXI1 feedback circuit and augment the efficacy of the chemotherapy drug temozolomide (TMZ) on inhibiting proliferation of malignant glioma cells (Xiao et al., 2020). In addition, the protease inhibitor nafamostat mesilate (NM) has been shown to inhibit the growth and metastasis of colorectal cancer and has anti-cancer effects in pancreatic cancer and lung cancer (Han et al., 2019b). Han et al. find that the activity of FTO can be inhibited by NM, indicating that NM might play a role in interfering with cancer progression as an FTO inhibitor. In addition, some biotech or pharmaceutic companies get down to developing high-efficiency small-molecule inhibitors, targeting the m6A regulators, in particular molecules like METTL3 and FTO that vary significantly in many cancers (Huang et al., 2020b). Undoubtedly, it provides a new choice for the effective management of a variety of cancers whether used alone or in combination with anti-tumour drugs, especially under the circumstance of chemotherapy drug resistance.

## 5 Conclusion and Prospects

Multiple regulation of various “writers” and “erasers” allows m6A modification in a dynamic and reversible manner, and significantly interacts with ncRNAs. Numerous studies have focused on the role of abnormal ncRNA m6A modifications in cancer progression. Aberrant m6A-modified ncRNAs and alteration of m6A regulators are closely related to cellular proliferation, apoptosis, invasion and metastasis, as well as other key steps of tumorigenesis and cancer progression. Undoubtedly, the cancers listed in this review form only a small part of the disease, and mainly include mainstream cancers and ncRNAs from recent years. Therefore, from the perspective of m6A modification, further research is needed to understand whether ncRNAs can participate in tumorigenesis and progression in other cancers, whether the same ncRNA plays the same role in different cancers (e.g. lncRNA MALAT1 and RBRP encoded by lncRNA LINC00266-1), and how different ncRNAs come into effect when they are involved in the same cancer; more research are also needed to explore the underlying mechanisms, especially in the field of stemness, reprogramming of tumour immune microenvironment and angiogenesis, and to illustrate the effects of ncRNA and m6A modification on cancer initiation, promotion and progression. Accordingly, the overall abundance of m6A modification may not be of vital significance; instead, some specific transcripts or genes play significant roles in biological function and cancer progression (most of them play the role in promoting cancer). Therefore, increased attention should be given to the significant changes in specific molecules, improving their clinical value as diagnostic/prognostic biomarkers. Further, studies now mainly focus on the cellular level, whereas fewer studies have been conducted *in vivo*. In clinical applications, several studies have elucidated the effect of ncRNAs in cancer prognosis based on m6A-Seq, m6A score, and designing data models, which has revealed molecules that could serve as biomarkers or therapeutic targets, and have greatly enriched the research in this field. However, applying these molecules in developing pharmaceutical preparations and in clinical diagnosis is still challenging. Overall, given the importance of ncRNA m6A modification and m6A regulators in various cancers, we consider that they are promising diagnostic and prognostic biomarkers contributing to the prediction of recurrence and survival, even serving as potential drug targets for therapeutic interventions in cancer treatment. Nevertheless, more specific mechanisms, more in-depth theories, and closer to practical applications of clinical diagnosis and treatment require further exploration.

## Abbreviations

CRC, colorectal cancer; ccRCC, clear cell renal cell carcinoma; ceRNA, endogenous ribonucleic acid; circRNA, circular ribonucleic acid; EMT, epithelial-mesenchymal transition; ESCC, esophageal squamous cell carcinoma; GC, gastric cancer; HCC, hepatocellular carcinoma; HNSCC, head and neck squamous cell carcinoma; lncRNA, long non-coding ribonucleic acid; miRNA, micro ribonucleic acid; m6A, N6-methyl-adenosine; ncRNA, non-coding ribonucleic acid; NM, nafamostat mesilate; NSCLC, non-small cell lung cancer; RBRP, RNA-binding regulatory peptide; 3ʹ-UTR, 3ʹ-untranslated region.
